# Challenges and Performance of Filter Dusts as a Supplementary Cementitious Material

**DOI:** 10.3390/ma17225676

**Published:** 2024-11-20

**Authors:** Johannes Berger, Anabella Mocciaro, Gisela Cordoba, Cecilia Martinefsky, Edgardo F. Irassar, Nancy Beuntner, Sebastian Scherb, Karl-Christian Thienel, Alejandra Tironi

**Affiliations:** 1 Institut für Werkstoffe des Bauwesens, Universität der Bundeswehr München, 85579 Neubiberg, Germany; johannes.berger@unibw.de (J.B.); nancy.beuntner@unibw.de (N.B.); sebastian.scherb@unibw.de (S.S.); 2 Centro de Tecnología de Recursos Minerales y Cerámica CETMIC (CONICET-UNLP-CICPBA), Manuel B. Gonnet B1897, Argentina; anamocciaro@cetmic.unlp.edu.ar; 3 Facultad de Ingeniería, CIFICEN (CONICET, CICBPA, UNCPBA), Olavarría B7400, Argentina; gcordoba@fio.unicen.edu.ar (G.C.); cmartinefsky@fio.unicen.edu.ar (C.M.); firassar@fio.unicen.edu.ar (E.F.I.); atironi@fio.unicen.edu.ar (A.T.)

**Keywords:** filter dust, recycling, supplementary cementitious material, SCM, cement-based composites, calcined clay, reactivity, physical properties

## Abstract

Global industry relies on a linear approach for economic growth. One step towards transformation is the implementation of a circular economy and the reclamation of anthropogenic deposits. This study examines two filter dusts, one German and one Argentinian, from the production of calcined clays, representing such deposits. Investigations and comparisons of untreated and calcined filter dust and the industrial base product pave the way for using waste product filter dust as supplementary cementitious material (SCM). In the future, some twenty thousand tons of contemporary waste could potentially be used annually as SCM. The results confirm the suitability of one material as a full-fledged SCM without further treatment and a measured pozzolanic reactivity on par with fly ash. Sample materials were classified into two groups: one was found to be a reactive pozzolanic material; the other was characterized as filler material with minor pozzolanic reactivity. Additionally, important insights into the physical properties of oven dust and heat-treated oven dust were obtained. For both material groups, an inversely proportional relationship with rising calcination temperatures was found for the specific surface area and water demand. The impact of the calcination temperature on both the particle size distribution and the potential to optimize the reactivity performance is presented.

## 1. Introduction

The construction and building industry contributes greatly to the world’s economy. In addition to the resulting increase in CO_2_ emissions, the consumption of vast amounts of primary resources presents a growing challenge for producers around the world, especially in densely populated areas like Germany. Due to an ever-increasing population on our planet and improving living standards, the demand for economic growth, while projected to be stagnant by 2040, will continue to be a driving force for the industry [1]. The OECD report estimates that by 2060 there will be an increase by 87% in material consumption in general and almost a doubling from 44 to 86 Gt for non-metallic materials, including cement and concrete [1]. Since the industrial revolution, humankind and its economic behavior relies more and more on a linear economy approach. This, also called the “take-make-waste” economy, according to the Ellen MacArthur foundation, runs the planet’s resources down with an ever-increasing rate that is currently linked to our population growth [2]. A decoupling of economic growth and the use of primary resources is needed and can be achieved only by changing the economic system to a circular approach.

Therefore, a careful assessment of primary and secondary resources is needed and urban deposits and possibilities in waste management especially need to be explored to enable better recycling strategies for the future. In addition to the well-known concept of utilizing materials from demolition sites, the concept of urban mining extends to all of the materials that have accumulated in the anthroposphere, including landfilled materials [3]. It describes all long-lived human goods, where long-lived is defined as a one-year minimum stay in a deposit of a relevant size [4]. While the possibility of prospecting landfills for valuable resources is a very important challenge, the circumvention of new waste materials and therefore the anticipation and capturing of tomorrow’s waste value is the key to a healthy future for humanity.

The Paris Agreement’s aim to limit “the temperature increase to 1.5 °C above pre-industrial levels” [5] is a focus of media and research. A popular approach to reduce CO_2_ emissions is the use of supplementary cementitious materials (SCMs) to achieve clinker-reduced binders [6]. The current market provides a variety of different SCMs, one of which is the group of calcined clays. The recent literature calculates the potential savings in kg-CO_2_ per m^3^ of concrete to be at least 16.5% for a 25% replacement of ordinary Portland cement (OPC) with an illitic shale and about 17.7% for the same replacement with a low-grade kaolin [7]. These findings are in line with Thienel and Beuntner, who calculate the potential savings in CO_2_ to be close to 15% [8]. In the near future, a lowered clinker factor will increase the demand for SCM [6]. Furthermore, established SCMs like fly ash and blast furnace slag will be of lesser importance due to the output limiting transformation in energy-intensive industries by 2030 as projected by [9]. While available in abundance and already in frequent use in some countries [10], calcined clays still play a minor role in the worldwide cement mix. This is going to change as more and more countries pass improved standards for wider varieties of cement mixes and, combined with the overall increasing demand for SCM, will boost the worldwide production of calcined clays to 2.6 Mt by 2030 [9,11].

However, while being potential materials to reduce CO_2_ emissions, they are still primary resources and thus a considerate waste-minimizing use is desirable. Another challenge is the availability of materials suitable for use as SCM close to a construction site, as transportation is a major cost and emission factor for the construction industry, especially in vast countries such as Argentina.

One possible solution for many of these challenges is the use of currently arising waste streams as future secondary resources to change from a linear to a circular approach. In the production of calcined clays on an industrial scale, a significant amount of dust is produced and discarded if not processed properly. The quantity and quality of the dust generated depends on the type of kiln employed and the nature of the clay. This issue is a hot topic for environment and health regulations in both Germany and Argentina.

On the German side, filter dust, although initially just a common clay, is legally considered a waste material due to being heat-treated in the industrial process [12]. It must therefore not be used to fill existing clay pits, but must be disposed of in special landfills, which takes up valuable landfill space and wastes large quantities of potential resources. However, §5 of the German circular economy law states that the declared waste character of a material ends when its suitability for a determined function is proven, there is a demand for it and it meets the respective standards and criteria to be safe for humans and nature [13]. One way to achieve this status is to fulfill the standards to be classified as a “supplementary building material” [12]. With this certificate, not only can precious landfill space be preserved and resources saved, but the respective company also saves a significant amount of money.

Although the Argentinian legal restrictions are different, recycled waste material still provides huge benefits due to the conservation of nature, increased product output, saved primary resources and short supply routes due to local production.

The aim of this paper is to assess two filter dusts that accumulate during the production of calcined clays in two industrial plants in Germany and Argentina for their suitability to be used as SCM. This project’s objective is to use the collected filter dust in combination with calcined clay in cement to contribute to the zero-waste ambition during calcined clay production. After determining the individual qualities, laboratory calcinations were performed to optimize the materials’ reactivities. Finally, raw and calcined filter dusts were compared to their respective industrial calcined clays in terms of their usability as SCM and real-world challenges for their use were identified.

## 2. Materials and Methods

### 2.1. Materials

The investigation of this international collaboration project focuses on two different filter dusts that are collected during the industrial production of calcined clay in two local production facilities in Germany and Argentina. Both facilities provided the individual research teams with one batch of each of the discussed samples. Those batches were around 20 kg on the German side and 100 kg on the Argentinian side. According to information from the suppliers, those batches resemble the current production processes that are under continuous monitoring to achieve consistent product quality on the industrial level. All samples were thoroughly mixed and subdivided to achieve homogenous laboratory samples for all the tests performed in this study. The German team cooperated with a producer of lightweight aggregates and calcined clay located in the state of Bavaria, Germany. A three-stage rotary kiln produces the calcined clay (CCC—calcined common clay) with an aimed material temperature of 750 °C [14]. The raw clay is fed to the kiln with rocks up to 100 mm in size, and the calcined material with a particle size of up to 40 mm is shipped to customers for milling. The total amount of collected filter dust comprises approximately 20% of the material throughput and is labeled D-CCC (dust of calcined common clay).

The Argentinian clay was obtained from a quarry near the city of Olavarría, Buenos Aires, Argentina. A single-stage rotary kiln with an aimed temperature of 950 °C produces industrial calcined clay (CIC—calcined illitic clay). The raw clay enters the rotary kiln with a particle size characterized by 11% residue on a 25.4 mm sieve and the calcined clay has roughly the same particle size. The shape of the material is that of laminated grain and the total amount of dust generated and collected is around 5% according to the production company. The collected filter dust is called DCIC (dust of calcined illitic clay).

The filter dusts calcined in the laboratory were designated with an additional “c” indicating calcination and the corresponding temperature (e.g., cD-CIC-750).

While the production temperature of both industrial products and the laboratory calcination is known, it has to be stated that it is not possible to measure the real temperature that the collected oven dust was exposed to.

### 2.2. Methods

#### 2.2.1. Chemical and Mineralogical Characterization

The chemical composition expressed as a percentage of oxides of the different samples (CCC, D-CCC, CIC, D-CIC) was determined via inductively coupled plasma–optical emission spectrometry (ICP-OES) according to [15] and described in detail in [16].

For the mineralogical characterization, X-ray diffraction (XRD) was performed in an Empyrean diffractometer, Malvern Panalytical Ltd., Malvern, Worcestershire, UK, using Cu-Kα radiation at 40 kV and 40 mA equipped with a BraggBrentano^HD^ Monochromator. Quantification was obtained through Rietveld refinement and the amount of amorphous phases was calculated with the internal standard method [17], using Profex BGMN [18].

Side-loading sample preparation with a particle size < 40 µm was used with 20 wt.% Zincite set as the internal standard. The identification of the mineral phases was achieved through an investigation of the 2 µm fractions on oriented mounts using the glass slide method [19]. The identification of swellable minerals was conducted on air-dried (AD) samples via treatment with glycol vapors in a 50 °C heated desiccator.

#### 2.2.2. Thermal Characterization

The optimal calcination temperatures for each filter dust were determined through thermal gravimetry (TG) (STA 449 F3 Jupiter), Netzsch-Gerätebau GmbH, Selb, Germany. Therefore, the dusts were heated up to 1000 °C with a heating rate of 10 °C min^−1^.

#### 2.2.3. Dust Calcination

Based on the thermogravimetric analysis, filter dusts were calcined at two different temperatures. The first one was set at the relevant maximum peak of the clays’ dehydroxylation temperature, the second one at 100 °C below it to test its suitability.

Due to varying facilities and practices in the laboratories of Germany and Argentina, the workflows to achieve the calcination differed. Both workflows are described in Table 1.

#### 2.2.4. Determination of the Calcination State

To assess whether the heat treatment achieved full calcination, Fourier Transformed Infrared spectroscopy (FTIR) was conducted at room temperature in attenuated total reflection (ATR) using a Nicolet iS10, ThermoFisher Scientific, Germering, Germany. The spectra were measured in the wavenumber range from 400 to 4000 cm^−1^ with diamond as the ATR crystal, collecting a series of 16 scans at a resolution of 4 cm^−1^.

#### 2.2.5. Assessment of Particle Properties

Particle properties were assessed using the particle size distribution (PSD), specific surface area, water demand and absolute density. PSD and its parameters (d_90_, d_50_ and d_10_) were determined using a laser diffraction analyzer Bettersizer S3 Plus, Bettersize Instruments Ltd., Liaoning, China, and Mie Scattering [20]. The specific surface area was measured with the BET method [21] using nitrogen gas with a Horiba SA-9600 Series. To determine the samples’ water demand, the Puntke method was applied [22]. The absolute density was measured using a ThermoFisher Scientific Pyknomatic ATC Helium pycnometer following the standard [23]. To facilitate comparisons with the existing literature, the location parameter x′ of the RRSB distribution was calculated [24].

#### 2.2.6. Reactivity Tests

The reactivity of the filter dust and calcined dust was determined with four different techniques: part one and two of the rapid, relevant and reliable (R^3^) test method [25], the Frattini test [26] and the solubility of Al and Si ions [27]. A comparison of the filter dusts with the industrially produced calcined clays (Argentinian calcined illitic clay (CIC), German calcined common clay (CCC)) allowed for an evaluation of the reactivity of the filter dusts.

Cumulative heat release, part one of the R^3^ test, was measured in a TAM Air isothermal Calorimeter, TA instruments, New Castle, DE, USA; chemically bound water, part two of the R^3^ test, was determined according to [25]. As a second way of ascertaining the reactivity, the Frattini test [26] was conducted at 7, 14 and 28 days. This provided additional information about the pozzolanicity of the material. According to DIN EN 196-5 [26], the Frattini Diagram is split by the curve of the maximum CaO solubility into two sections: everything below the curve passes the test; everything above does not. To facilitate reading and comparison, the coefficient *X_Fr_* based on [28] was introduced. *X_Fr_* represents the vertical distance of the measured data point to the solubility curve, calculated according to Equation (1). The given variables express the isothermal curve of the maximum calcium ion solubility expressed as calcium oxide (lime solubility curve) [26] (${\left[CaO\right]}_{MAX}$), the calcium concentration of the sample (${\left[CaO\right]}_{Sample}$) and the given hydroxide concentration that describes the lime solubility curve.

(1) $$X_{Fr}=\frac{350}{[OH]-15}-{\left[CaO\right]}_{Sample}={\left[CaO\right]}_{MAX}-{\left[CaO\right]}_{Sample}$$

After this calculation, higher positive values signify higher pozzolanic reactivity.

The solubility of silicon (Si) and aluminum (Al) ions in alkaline solution was measured via ICP-OES, using a Varian 720 ES spectrometer according to [27]. External standards and a multi-point calibration were used according to [15]. To dissolve the ions, the preparation routine described in [29] was performed.

## 3. Results and Discussion

### 3.1. Chemical Characterization of Materials

The chemical composition (Table 2) of the filter dust D-CCC and the raw material CCC shows minor differences. The content of SiO_2_, Al_2_O_3_ and CaO is reduced by 1.1 to 3.3 wt.% in the D-CCC compared to CCC, whereas the content of Fe_2_O_3_ and SO_3_ is slightly higher, with 2.3 and 1.3 wt.%. The D-CCC shows almost double the LOI compared to CCC.

The chemical composition of the Argentinian filter dusts (D-CICs) and the corresponding industrial products (CICs) differ further. A significantly higher SiO_2_ content of 7.4 wt.% in CIC and reduced contents of CaO and Na_2_O can be observed. The thorough calcination of the industrial product results in a lower LOI by 5.3 wt.% compared to the only partially calcined dust. All samples in Table 2 comply with the limits for the LOI, SO_3_ and CaO for Class N Natural Pozzolans described in ASTM C618-23 and all, except the D-CIC, comply with the limits for Class F Natural Pozzolans [30]. While it is proven that this standard allows for false positives, as elaborated in [31], the assessment of the reactivity in Section 3.4 clarifies this question and complying with the standard makes practical use easier in construction applications.

### 3.2. Mineralogical Characterization of Materials

The German sample originates from the Lias delta clay formation of the lower Jurassic in the region of Nuremberg [32,33]. It therefore has a high content of clay minerals, with mica and kaolinite making up more than 50% of the material.

On the contrary, the raw material of the Argentinian filter dust comes from a region where the “Sierras Bayas Group-Cerro Negro Formation” [34] is the prevalent geological structure. The main clay minerals of the raw material are illite, chlorite, smectite and fine granular quartz as an impurity [35]. These geological differences are reflected in the mineralogical composition as shown in Table 3. In general, a quantification of (partly) calcined samples poses great challenges, but is important to obtain an idea of the share of uncalcined clay and swellable clay minerals.

The D-CCC reveals a lower amorphous content by ~32 wt.% in comparison with CCC. Given the actual calcination temperature of CCC (~750 °C), glass formation and the calcination of the carbonates can be ruled out. Thereby, the difference in the content of clay minerals explains the variation in the amorphous content. The sample D-CCC contains 33 wt.% more of different phyllosilicates, which, within a margin of error, can be attributed to the higher amorphous content of CCC. However, the content of quartz, hematite and anhydrite is lower in the filter dust, which, in general, points towards a slight segregation of different particles owing to the industrial process as discussed in Section 2.2.5. Due to the smectite being bound in an illite/smectite alternation, neither the D-CCC nor CCC contains any swellable clay minerals [36].

**Figure 1:**
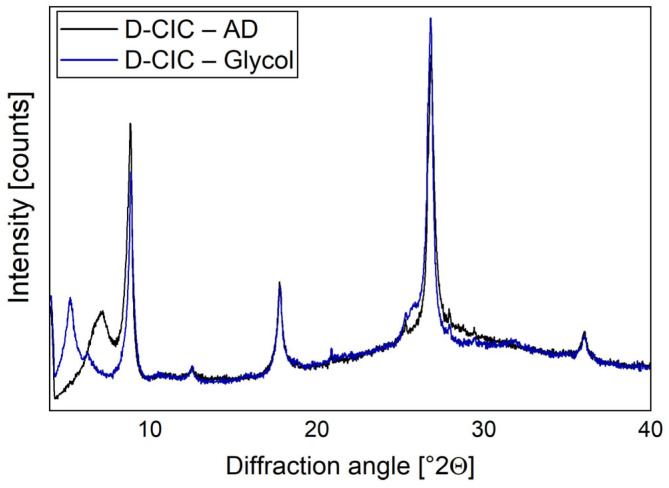
XRD assessment of swellable clay minerals in D-CIC through glycol vapor treatment. D-CIC—AD represents the air-dried and D-CIC—Glycol the glycol vapor-treated D-CIC sample.

During the analysis of the Argentinian samples, the distinction between smectite and chlorite required a glycol vapor treatment described in Figure 1. Figure 1 shows the resulting shift in the XRD peaks due to the widened layers after treatment.

**Figure 2:**
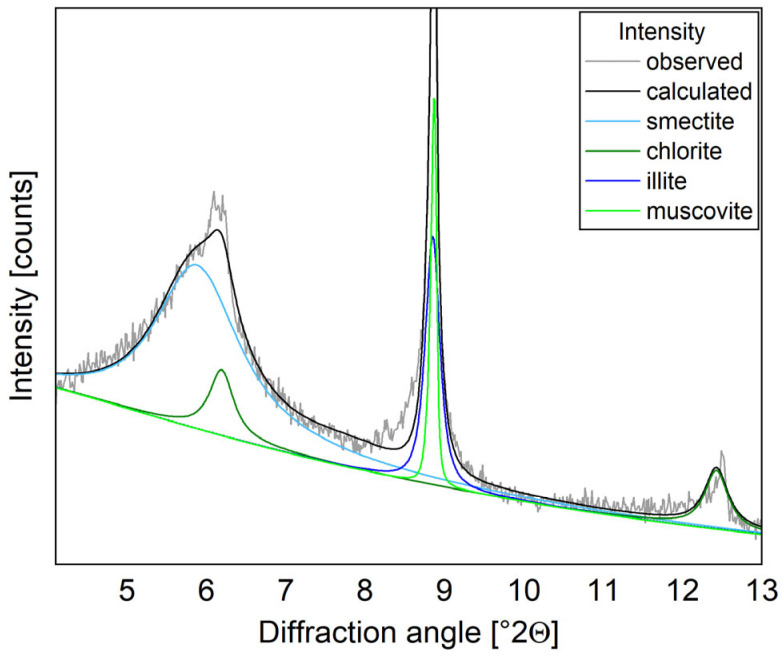
XRD segment of the XRD quantification showing the different phyllosilicates present in the D-CIC.

The peak at 6 °2θ with an attached shoulder can be identified as a combination of chlorite and swellable smectite (Figure 2). This facilitates a better overall fit during the Rietveld refinement and allows for a confident final quantification.

The detailed analysis of the mineralogical composition of the Argentinian samples indicates a different link between the product (CIC) and the collected dust (D-CIC). In contrast to the 17 wt.% higher amorphous content in the CIC sample, the portion of the phyllosilicates is much lower with a combined total of ~−30 wt.%, whereas the content of quartz is 11 wt.% and that of feldspar is 3 wt.% higher than in the collected dust. Thus, it appears that the segregation effect in the production process of CIC is much stronger than in the German facility.

A possible explanation for the segregation effects in both samples is the nature of the production process, in which a strong airflow supports the combustion of the fuels in the kiln. The airflow is likely to favor the transport of fine and light particles, thus enriching them in the dust to a certain degree. Those particles are likely clay minerals due to their lower hardness and ability to withstand deagglomeration compared to the structural integrity of the bigger and harder quartz and feldspar particles.

### 3.3. Impact of Temperature on Properties of Filter Dust

**Figure 3:**
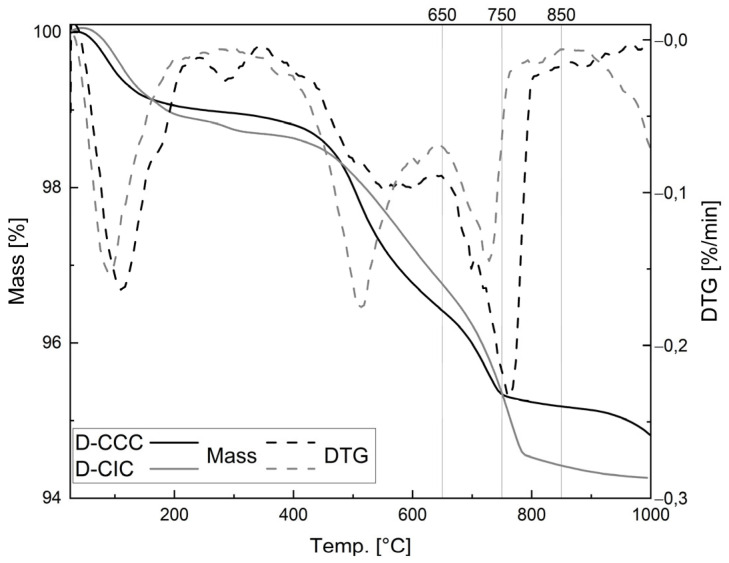
TG and DTG curves of the two dusts D-CCC and D-CIC with marked calcination temperatures.

Figure 3 shows the thermal analysis of the two filter dusts. The different stages of lost mass are detailed for both filter dusts in Table 4. The D-CCC sample exhibits three stages: The first one below 300 °C is related to the water desorption and bound interlayer water. The second stage between 300 and 650 °C characterizes the dehydroxylation of kaolinite, and the last stage up to 1000 °C is associated with the dehydroxylation of illite and the decarbonation of the minor carbonate content with a mass loss of 1.60% [37]. In total, that amounts to a mass loss of 5.18%.

On the other hand, four different stages can be distinguished for the D-CIC sample. The first area, below 250 °C, is associated with the water adsorption and the dehydration of the interlayer water of smectite [38]. The second stage in the range of 250–350 °C indicates either the loss of interlayer water from T-O-T clay minerals [35] or the composition of iron (III) oxide-hydroxide [39]. From 350 to 650 °C and between 650 and 1000 °C, the dehydroxylation of illite, smectite, muscovite, and chlorite takes place in two steps with a mass loss of 1.93% and 2.50%, respectively [37]. This leads to a total loss of mass of 5.74%.

Kaolinite has reached ~95% dehydroxylation already at 650 °C according to [37], compared to the temperature of up to 1000 °C that is needed to calcine illite, smectite and chlorite. Thus, an increased amount of kaolinite in a material lowers the temperature required to reach a certain degree of dehydroxylation. Consequently, the calcination temperatures for the D-CIC, shown in Figure 3, were set to 750 and 850 °C (cD-CIC-750 and cD-CIC-850, respectively), and for the D-CCC to 650 and 750 °C, accounting for the kaolinite content (cD-CCC-650 and cD-CCC-750, respectively).

The actual calcination was performed according to Section 2.2.3.

#### 3.3.1. Degree of Dehydroxylation

**Figure 4:**
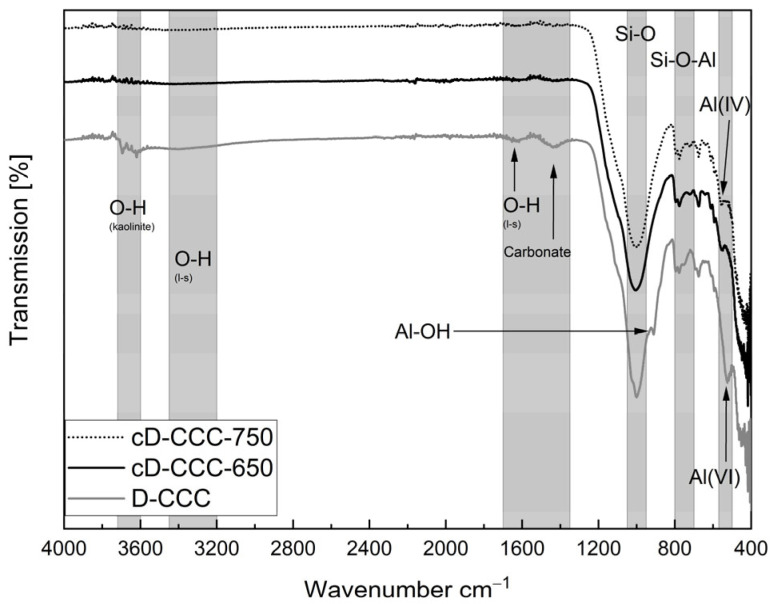
FTIR spectra, comparing German filter dust before and after treatment.

**Figure 5:**
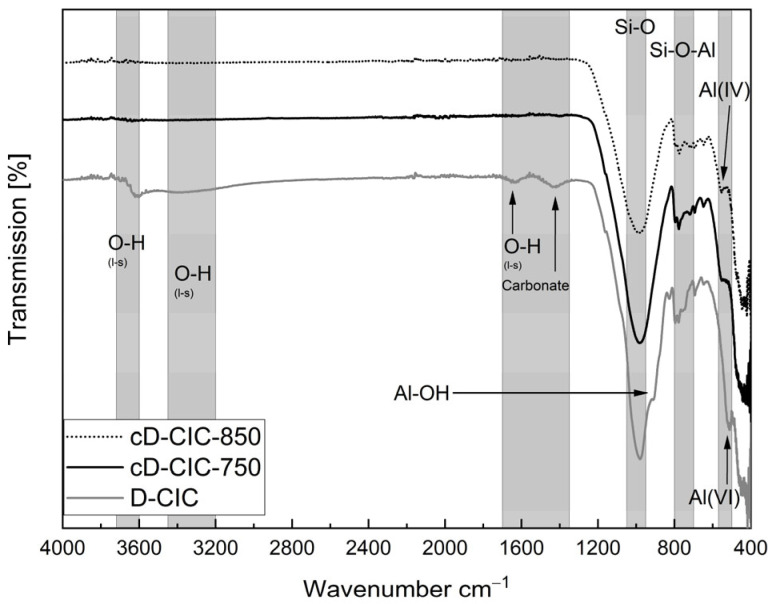
FTIR spectra, comparing Argentinian filter dust before and after treatment.

Figure 4 and Figure 5 display the FTIR spectra of the two filter dusts and the heat-treated sample materials. The gray columns indicate the different wavenumber areas corresponding to the individual markers relevant for assessing the calcination process according to [37,40,41]. The LOI and XRD display the amorphous content and indicate partly calcined filter dusts. However, the FTIR spectra still show a significant portion of uncalcined phases in both material groups and therefore a potential to increase the reactivity of the samples.

After calcination at 650 °C, the FTIR spectra of the D-CCC samples no longer show the characteristic stretching bands of the OH groups of kaolinite between 3620 and ~3695 cm^−1^ as well as the other phyllosilicates at ~3300 cm^−1^, ~1620 cm^−1^ and ~1420 cm^−1^. However, the Si-O band intensity at 1000 cm^−1^, as well as the two alumina bands, is not reduced, and the secondary Al-OH band at 911 cm^−1^ does not yet disappear until 750 °C is reached [37,40,41].

The characteristic sections of the OH groups are completely gone after calcination at 750 °C in the three spectra of the Argentinian material group (Figure 5). The important areas are in this case 3600–3700 cm^−1^, ~3400 cm^−1^ and ~1640 cm^−1^ and can be attributed to the OH groups of the different phyllosilicates, abbreviated with “l-s” in the diagram. The band at ~1420 cm^−1^ indicates carbonate [42]. The absence of all the aforementioned peaks confirms a full dehydroxylation of the samples. However, the disappearance of the Al-OH shoulder at 909 cm^−1^ and the reduction in the Si-O peak intensity at 980 cm^−1^, described by [43], as well as the two alumina sections, are not completed until 850 °C.

#### 3.3.2. Physical Properties

Table 5 presents the physical properties of the different materials. For the particle size distribution, the corresponding d_10_, d_50_ and d_90_ values for each of the investigated samples are listed, as well as the location parameter x’ of the RRSB distribution. The sample group associated with the D-CCC shows a significant trend of coarsening particle size distribution with higher calcination temperatures. From the untreated D-CCC to the cD-CCC-750, a gradual increase in particle size is observed. For d_50_ and d_90_, this has considerable values of 25 and 32%, respectively.

The samples associated with the D-CIC have a somewhat different tendency: While exhibiting an increasing particle size with a rising calcination temperature for the d_10_ and d_50_ values_,_ with the D-CIC having the lowest and the cD-CIC-850 the highest, the results for the d_90_ values are different. The highest particle size is observed in the cD-CIC-750, with the D-CIC and the cD-CIC-850 both having 4% and 6% lower values, respectively. However, all values lie within a narrow range.

The general idea of a coarsening particle size with rising calcination temperatures is already described in [44] for low-grade kaolinitic clays with kaolinite, illite and montmorillonite as the main clay minerals. Apart from the d_90_ values of the D-CIC sample group, all measured samples fit this tendency. Interestingly, the D-CIC also matches these results, although it does not contain kaolinite. Therefore, it seems that this perception can generally be transferred to clay mineral-containing materials.

While comparing the filter dusts to their industrial counterparts, it must be pointed out that CCC and CIC are produced as coarse-grained aggregates with particle sizes of up to 40 mm (CCC) and 25 mm (CIC). Grinding is performed in a separate step, optimizing the PSD for use in cement, while the filter dusts were tested in their unground form. In comparison, both groups of filter dusts are much coarser than the ground industrial materials. The d_50_ of CCC is only 60–80% of that of the filter dust samples. The measured d_90_ is only 40–60% of that of the D-CCC and its heat-treated derivates. Similar results were obtained from the Argentinian samples. CIC’s d_50_ is 72–84% and the d_90_ 50–54% of that of the D-CIC samples.

Table 6 shows the PSD ranges of three materials, described by the location parameter x′ as the 63.2% quantile of the RRSB distribution [24] obtained from the literature. These parameters can be compared to the data in Table 5. The fineness of the untreated filter dusts, the D-CCC with 30.0 µm and the D-CIC with 30.5 µm, compares well to the cement presented in Table 6. Although this does not hold for the other samples with bigger diameters, they still are comparatively close to the established SCM and to that of CEM I 32.5 R. Based on the PSD, all analyzed filter dusts, uncalcined as well as calcined, are suitable to be used in blended cements without further grinding.

The particle densities of the untreated filter dusts, the D-CCC as well as the D-CIC, are 2% higher than the industrial end product. This corresponds with the presumed lower density associated with the loss of hydroxyl groups during the calcination process [48]. The lab calcination of the individual dusts resulted in a slight variation in the individual densities. With higher calcination temperatures, they appear to decrease for the Argentinian samples, whereas for the German samples, they increase. However, those changes are fairly small and all within <1% of the standard deviation.

The BET surface area of the D-CCC samples of 18.5 m^2^/g decreases to 14.3 m^2^/g for the cD-CCC-750 with an increased calcination temperature. Interestingly, CCC presents itself with a much lower BET surface area, with only 3.8 m^2^/g. This difference between the D-CCC samples and CCC can neither be explained by the temperature nor by the deviating PSD. If the production process is examined closely, it can be split into different individual parts for the samples. While CCC is calcined in a rotary kiln, the influence of the dust separation in the hot process gas might be comparable to the effects seen in flash and fluidized bed processes. Due to the mechanical deagglomeration of the material in a rotary kiln, the exposure time in the oven is much longer than the 5 s one in a fluidized bed and even more than that in a flash calciner according to [49], but still significantly shorter than the 30 min stated by the manufacturer for CCC. The X-ray amorphous content of the D-CCC (Table 3) indicates that the residence time and temperature in the gas stream have a long resp. high enough to calcine part of the kaolinite. In this regard, the difference in the BET surface area between the heat-treated dusts might be due to the different calcination mechanisms. Hanpongpun et al. compared two clays of a similar median particle size that had been calcined in different processes [49]. They found an 18% higher BET surface area for fluidized bed calcination compared to rotary kiln production.

The sample group with the D-CIC exhibits a different behavior, which can be attributed to the raw material not containing kaolinite and the time in the hot process gas not being sufficient to calcine the present clay minerals. These findings are backed up by [33,50,51], who describe in depth the change in the BET surface area with a varying calcination temperature.

Contrary to the specific surface area of the filter dusts, the water demand determined with the Puntke method increases with rising calcination temperatures. While this increase is 30% from the uncalcined sample to the material calcined at 750 °C for the D-CCC, it is still a 60% lower water demand compared to CCC. Again, the same tendency was observed for the D-CIC with an increase of 53% from the uncalcined dust to the sample calcined at 850 °C (cD-CIC-850). However, the difference between CIC and the D-CIC is only 32%.

**Figure 6:**
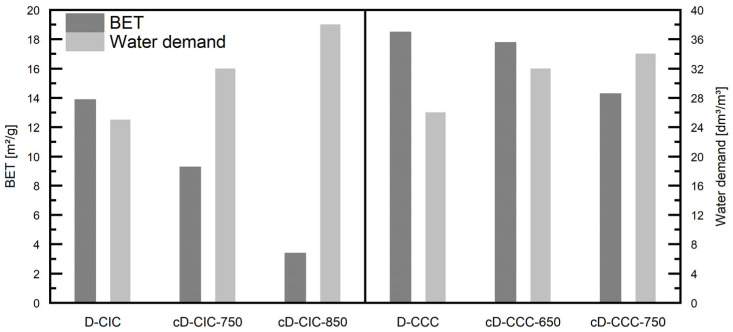
Visualization of the inverse proportional relationship between the BET surface area and the water demand determined with the Puntke method with qualitatively increasing temperatures for both of the investigated sample groups.

Summarizing the measurements of the physical properties, four significant tendencies for both sample groups are identified to facilitate the subsequent discussion. One is the significantly higher BET surface area of the filter dusts compared to the industrial products. A trend contrary to this finding is the much lower water demand determined with the Puntke method of the D-CCC and the D-CIC at the mentioned higher BET surface area compared to the industrial product. Furthermore, with an increasing calcination temperature, both a decreasing BET surface area and increasing water demand have been observed. This inversely proportional behavior is visualized in Figure 6. It has to be mentioned that the horizontal axis, while showing the different calcination temperatures, has to be seen in a qualitative way. As mentioned in Section 2.1, the temperature to which the oven dust was exposed could not be measured in the industrial process. However, due to the FTIR analysis elaborated in Section 2.2.4, it is certainly below that of the calcined dusts, hence the order shown in Figure 6.

The measurements concur very well with the findings [52], where a similar behavior is presented for (calcined) muscovite. It states that not only might the BET and PSD contribute to these findings but also the mineralogy and the calcination process itself.

However, He et al. investigated the water/(cement + clay) ratios in mortars for six different clays to obtain 100% flow as well as the BET surface area for the different calcination temperatures of the individual clays [53]. Of the six clays, four exhibited a drastic reduction in the BET surface area and water demand in the corresponding mortars with an increasing temperature. The other two mixtures showed similar, albeit smaller, effects.

Besides the influence of the mineralogical composition, another explanation for the inversely proportional relationship between the BET surface area and water demand hinges on the physical parameters. The observed coarsening effect as well as particle agglomeration might present a reason for the observed behavior. During the heat treatment, the formation of agglomerates of different sizes was observed, despite temperatures far below any melting point. This indicates a formation of robust and compact particle clusters and provides a plausible explanation for the anti-proportional effect of a decreasing BET surface area and increasing water demand.

The BET method measures the surface area by applying a multimolecular layer of the test fluid, in this case nitrogen, on the surface of the sample particles [54]. While the coarsening of particles, formation of compact clusters and potential closing of small pores decrease this area, the bulk density of the material stays the same or even declines due to the increased particle size. In contrast to the multilayer mechanism of the BET method, the Puntke test aims to fill all voids present in the bulk of the sample right to the point where liquid shows on the surface of the sample [22]. A lower bulk density therefore means more voids that can be filled with water, and thus a higher water demand determined with the Puntke method.

### 3.4. Reactivity of Untreated and Calcined Filter Dust

Due to the different geological structures the raw material originates from (Section 3.1), the mineralogical composition and therefore the expected reactivity in both the untreated and heat-treated filter dusts differ.

Table 7 and Table 8 provide the results of the four methods that were used to assess the reactivity of the different materials, namely the two parts of the R^3^ test (accumulated heat and chemically bound water), the Frattini test and the solubilities of Al and Si ions.

The results of the two parts of the R^3^ test correlate well for the German sample group and indicate the D-CCC as having the lowest and CCC the highest reactivity of this cluster. The Argentinian samples present a different picture with the cD-CIC-850 having the highest reactivity and that of the industrial product CIC being almost 20% lower. The standard deviation for the bound water test according to the R^3^ method was calculated to be between 1.6% (CCC) and 5.3% (D-CCC). In general, the Frattini test supports these results. However, the ranking of the individual samples and the relative degree of reactivity do not correspond well with the R^3^ test outcome. This discrepancy is in line with the results of RILEM TC 267-TRM [55], where the R^3^ test is described as the most reliable test for pozzolanic activity. The possible conclusions based on the combined solubility of Si and Al ions are more in line with the results of the R^3^ test. This does hold except for CIC, which has the highest solubility of the Argentinian samples, and the D-CCC, which, unlike in the R^3^ test, has a solubility higher than the cD-CCC-650.

**Figure 7:**
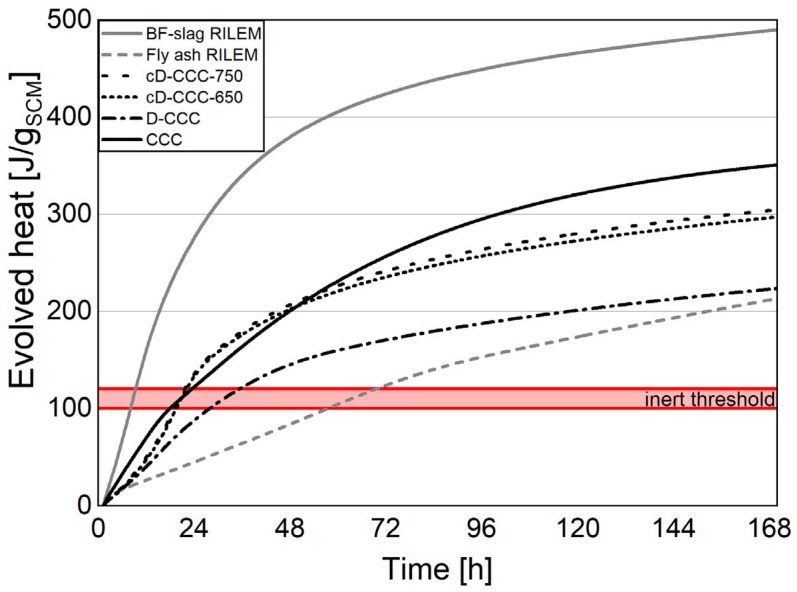
R^3^ test for evolved heat; German samples with reference curves and inert threshold band according to [9].

**Figure 8:**
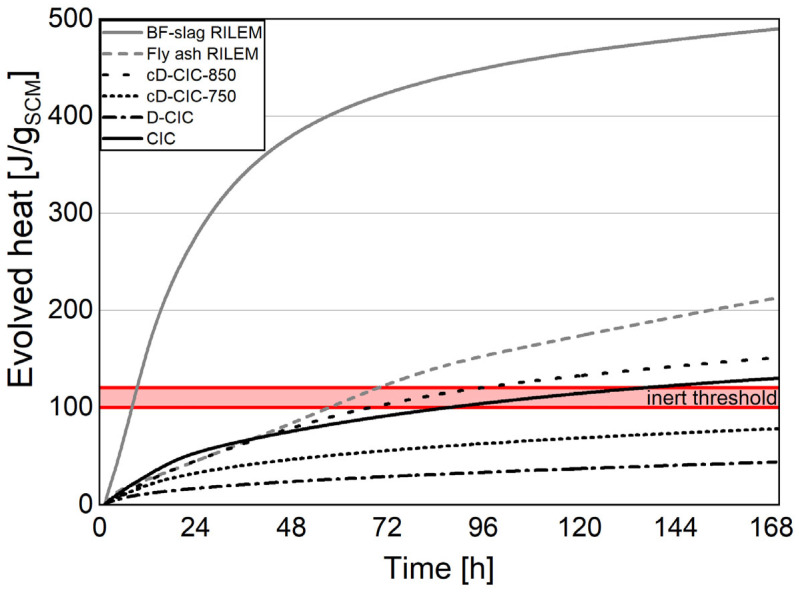
R^3^ test for evolved heat; Argentinian samples with reference curves and inert threshold band according to [9].

Figure 7 and Figure 8 emphasize this further by showing the heat release of the individual samples together with the references fly ash and blast furnace slag adapted from [55,56]. Further, the aforementioned threshold to distinguish between reactive and inert material is adapted from [9] and displayed as a red horizontal band stretching from 100 to 120 J/g_SCM_.

The added information helps classify the results into two different material groups, as was already suspected from the other methods of characterization.

The reactivity of the D-CCC is well above the inert threshold and on par with the fly ash at the end of the seven-day test period. However, the two post calcinations could not achieve the reactivity of the original product, CCC. This may be explained by two different factors. First, although the aimed temperature in the production process is 750 °C, it could be somewhat higher since it is very difficult to determine the actual material temperature in practice. Infrared thermometers do not work well due to the dusty kiln atmosphere developed during the process. The additionally practiced control of the process temperature on manually collected samples at the head of the rotary kiln underestimates the actual calcination temperature due to the rapid cooling of the sample in the open air. Furthermore, the industrial product is ground, as detailed in Section 3.3.2, and specifically optimized for use in cement. In contrast, the different states of the treated and untreated filter dusts were processed without further grinding, which can affect the reactivity dramatically, especially during the first seven days.

The reactivity of the samples associated with the D-CIC is below the reference fly ash and they must, in most cases, be considered as inert filler materials. Interestingly, the CIC sample does not have the highest reactivity of the Argentinian samples, because of the segregation effect, which was already described in Section 3.1. Due to the higher quartz and lower combined clay mineral content in CIC compared to the D-CIC, there is less thermal activation potential.

### 3.5. Implication Regarding the Use of Filter Dusts as SCM

There are a number of quality requirements linked to the potential use of filter dust from the production of calcined clays as SCM. The first requirement is an adequate chemical and mineralogical composition. Following the results in Section 3.1, the chemical composition complies with the ASTM C618-23 limits for Class F Natural Pozzolans for three out of four discussed samples (D-CCC, CCC, D-CIC and CIC), with only the D-CIC belonging to the less strict Class N Natural Pozzolans [30].

Both filter dust groups contain high amounts of uncalcined clay minerals and some amorphous content. High amounts of swellable clay minerals such as smectites were measured in the D-CIC, which can be harmful in concrete, especially concerning freeze–thaw resistance. Since there is no standard for uncalcined clays in cement, alternative standards are consulted to sketch a picture of the current limitations in general. DIN EN 12620 regulates the parameters of swellable clay minerals in the fine fractions of aggregates, often in the form of clay minerals [57]. If a fine aggregate with a diameter smaller than 8 mm contains more than 3 wt.% fines, these fines need to be tested for quality either by determining a sand equivalent or a methylene blue value [57]. The fine aggregate content in a normal concrete mix usually is about 400–500 dm^3^/m^3^, which means an allowed maximum of untested fines of 15 dm^3^/m^3^. Assuming a typical binder content of 110 dm^3^/m^3^ and a moderate replacement of 20 wt.%, the share of filter dust would already amount to 22 dm^3^/m^3^. Therefore, a quality check would be inevitable.

The DTG measurements provide information about the temperatures required to optimize the potential of both filter dusts. The different calcination stages were observed via FTIR and the degree of calcination as well as the risk of the presence of the swellable clay minerals could be analyzed. The results confirm the complete calcination and dehydroxylation of all potentially harmful uncalcined particles only for the cD-CCC-750 and the cD-CIC-850. These two calcined filter dusts would not need further assessment according to DIN EN 12620 [57].

While the PSD of the raw and calcined dusts differ from that of the industrial end products, they are still close to that of current SCM and binder components as shown in Table 5 and Table 6. Further tasks will be a weighing up of benefits regarding the efficiency as a filler and reactivity properties on the one hand, with added costs, energy demands and therefore additional emissions being calculated in the life cycle assessment (LCA) on the other hand. In addition to the considerations regarding the filler effect and reactivity linked to the PSD, a tendency was identified considering the BET surface area and water demand. While the lower water demand of the filter dusts compared to their corresponding industrial products might be beneficial for their use in a binder, the much higher SSA could present other difficulties. Recent findings [58] show a correlation for LC^3^ systems between a higher BET of calcined clays in these binder systems and an increased demand for superplasticizers. This, plus the proportion of uncalcined phyllosilicates in some of the samples discussed here, might pose a challenge to adjusting the flow of binder systems with filter dusts as these phyllosilicates are known to absorb conventional PCEs [59].

## 4. Conclusions

This paper presents two filter dusts from production facilities for calcined clays that were collected to assess their potential use as SCM and further means of optimizing their potential. A general realization of this project is that despite belonging to the same material group, oven dusts vary widely regarding their mineralogical and physical properties. Due to different raw materials and production conditions, solutions for their use need to be tailored to the individual material. Thus, a wider investigation of different kiln dusts from different plants and regions is indispensable. It is necessary to investigate whether the main insights of this publication apply to other filter dusts.

The investigation of the two aforementioned filter dusts led to the following findings:

- The results show a clear segregation effect for both of the filter dusts in comparison to industrially calcined clays. This is less pronounced for the D-CCC compared to the D-CIC, especially with regard to the enrichment of phyllosilicates and the decrease in the quartz and feldspar content. It is assumed that the airflow in the rotary kiln and the deagglomeration due to the rotation and impact of the aggregates cause the segregation and thus smaller and lighter clay particles are more easily entrained into the airflow.
- The samples can be classified into two groups according to their reactivity. Those associated with the D-CCC can be classified as reactive supplementary cementitious material, while the ones associated with the D-CIC could pose as filler materials with slight pozzolanic properties.
- The cD-CCC-750 presents lower reactivity than CCC.
- The cD-CIC-850 has higher reactivity than CIC, which might be attributed to the enrichment of clay minerals in the D-CIC due to a pronounced segregation effect.
- Both filter dust groups exhibit a significantly lower water demand than their corresponding industrial products, while the further calcination of both filter dusts increases the water demand with an increasing calcination temperature.
- Both filter dust groups have a significantly higher BET compared to their corresponding industrial products, while the further calcination of both filter dusts reduces the BET surface area with an increasing calcination temperature.
- The coarsening of particles and formation of clusters due to the calcination of filter dusts might increase the void volume within the dust particles, which could explain the inversely proportional effect observed regarding the decreasing BET surface area and increasing water demand.

Because of its reactive properties, direct use in cement is possible for the D-CCC, while the D-CIC qualifies as filler material with slight pozzolanic potential. However, several problems that need to be solved arise: first, the assessment of potentially harmful uncalcined swellable phyllosilicates that might impact the freeze–thaw resistance; second, the significantly increased BET surface area that, although the water demand drops, might impact the consumption of PCEs. These questions and a general evaluation concerning the LCA and mechanical properties of mortars will be addressed in the next phase of the project.

## Figures and Tables

**Table 1:** Calcination workflow.

Argentina	Germany
Place the samples in a shallow container in the cold oven. Heat oven to target temperature (~10 °C min^−1^). Keep temperature constant for 1 h. Let oven cool down naturally with closed door and sample inside.	Heat oven to desired temperature. Place the sample in a shallow container inside the preheated oven. Keep temperature constant for 1 h. Remove sample and let cool down in desiccator.

**Table 2:** Chemical composition of the calcined clays in wt.%.

	Filter Dust		Industrial Products	
Sample	D-CCC	D-CIC	CCC	CIC
SiO_2_	50.6	53.9	51.74	61.3
Al_2_O_3_	18.3	16.3	21.6	16.4
CaO	5.2	3.9	6.5	2.2
Fe_2_O_3_	10.3	6.8	8.0	6.8
K_2_O	2.4	4.3	2.9	4.3
MgO	2.2	2.2	2.9	2.2
Na_2_O	0.9	5.8	0.8	4.2
TiO_2_	1.3	0.8	1.0	0.9
SO_3_	3.1	0.2	1.8	0.3
LOI	5.5	6.7	2.8	1.4

**Table 3:** Mineralogical composition of the materials in wt.%.

	D-CCC	CCC	D-CIC	CIC
Quartz	16	19	21	32
Carbonate	3	3	3	-
Chlorite	3	-	10	1
Pyrite	1	-	-	-
Feldspar	4	3	12	15
Muscovite	6	-	-	-
Illite/Muscovite	-	-	33	21
Illite/smectite alternation	36	22	-	-
Smectite	-	-	15	8
Kaolinite	10	-	-	-
Rutile/Anatase	3	-	1	1
Hematite	-	2	-	-
Anhydrite	-	2	-	-
Amorphous	17	49	5	22

**Table 4:** Thermal parameters and mass loss of D-CIC and D-CCC samples.

Sample	Temperature Range	Loss of Mass (%)	Maximum in DTG Curve (°C)
D-CCC	25–300	1.03	89.3
	300–650	2.55	505.5
	650–1000	1.60	721.7
D-CIC	25–250	1.12	106.8
	250–350	0.19	292.3
	350–650	1.93	579.9
	650–1000	2.50	764.2

**Table 5:** Physical properties of the raw and calcined filter dusts and the industrial products.

	D-CCC	cD-CCC-650	cD-CCC-750	CCC	D-CIC	cD-CIC-750	cD-CIC-850	CIC
Particle size (µm)								
d_10_	2.3	2.5	2.8	4.0	2.4	3.0	3.9	2.1
d_50_	19.1	21.7	23.9	13.2	19.7	21.8	22.8	16.5
d_90_	77.2	88.6	101.9	37.0	90.0	93.8	88.2	47.7
x′ (63.3%) RRSB	30.0	34.5	38.0	16.7	30.5	33.2	33.8	23.3
Density (g/cm^3^)	2.65	2.66	2.66	2.60	2.75	2.71	2.70	2.69
BET (m^2^/g)	18.5	17.8	14.3	3.8	13.9	9.3	3.4	4.6
Water demand (dm^3^/m^3^)	26	32	34	42	25	32	38	33

**Table 6:** PSD of cement and SCM described by x’ after RRSB [24].

Sample	RRSB: x′ (63.2% Quantile)
CEM I 32.5 R [45]	19.6–31.3
Fly Ash [46]	26.5
EFA Filler (Electro-Filter Ash) [47]	21.8

**Table 7:** Reactivity, measured by R^3^ test and Frattini test.

	R^3^ Calorimeter	R^3^ Bound Water	Frattini
Sample	Accumulated Heat Over 168 h [J/g_SCM_]	Bound Water After 7 d (→168 h) [%]	*X_Fr_*—Pozzolanic Coefficient (28 d) → See Section 2.2.6
D-CCC	222	5.21	3.39
cD-CCC-650	296	6.71	2.96
cD-CCC-750	304	6.71	3.46
CCC	349	8.11	4.09
D-CIC	43.7	2.00	−0.17
cD-CIC-750	78	2.40	0.34
cD-CIC-850	151	3.70	0.06
CIC	129	3.10	1.15

**Table 8:** Solubilities of Al and Si ions.

Sample	Al [mmol/L]	Si [mmol/L]	Al + Si [mmol/L]	Si/Al
D-CCC	0.95	1.25	2.20	1.31
cD-CCC-650	0.67	0.82	1.49	1.23
cD-CCC-750	1.53	2.00	3.53	1.31
CCC	1.44	2.40	3.84	1.67
D-CIC	0.11	0.27	0.38	2.46
cD-CIC-750	0.32	0.69	1.01	2.19
cD-CIC-850	0.31	0.80	1.11	2.56
CIC	0.41	0.89	1.30	2.18

## Data Availability

The original contributions presented in the study are included in the article, further inquiries can be directed to the corresponding author.
